# Supplementary material for: Unveiling the Bioactive Potential of the Invasive Jellyfish Phyllorhiza punctata Through Integrative Transcriptomic and Proteomic Analyses
Source: Biomolecules. 2025 Aug 4;15(8):1121. doi: 10.3390/biom15081121 (PMC12383608; doi:10.3390/biom15081121)
Supplement: Supplementary file 1 [file biomolecules-15-01121-s001.zip › Suplementary Material/FileS3/PEPFOLD4/XP_065067185_14731/clusters.html]

 PEP-FOLD clusters


Download archive of all models  here   

|  |  |  |
| --- | --- | --- |
| # | file | sOPEP |
| # cluster 1 - Size: 1 |  |  |
|  | XP\_065067185\_14731-model1 | -63.3155 |
| # cluster 2 - Size: 1 |  |  |
|  | XP\_065067185\_14731-model2 | -61.3052 |
| # cluster 3 - Size: 1 |  |  |
|  | XP\_065067185\_14731-model3 | -59.039 |
| # cluster 4 - Size: 1 |  |  |
|  | XP\_065067185\_14731-model4 | -59.0074 |
| # cluster 5 - Size: 1 |  |  |
|  | XP\_065067185\_14731-model5 | -55.8379 |
| # cluster 6 - Size: 1 |  |  |
|  | XP\_065067185\_14731-model6 | -52.4421 |
| # cluster 7 - Size: 2 |  |  |
|  | XP\_065067185\_14731-model7 | -52.1612 |
|  | XP\_065067185\_14731-model7.1 | -45.7025 |
| # cluster 8 - Size: 1 |  |  |
|  | XP\_065067185\_14731-model8 | -51.3956 |
| # cluster 9 - Size: 1 |  |  |
|  | XP\_065067185\_14731-model9 | -50.952 |
| # cluster 10 - Size: 1 |  |  |
|  | XP\_065067185\_14731-model10 | -50.3108 |
| # cluster 11 - Size: 1 |  |  |
|  | XP\_065067185\_14731-model11 | -49.6438 |
| # cluster 12 - Size: 1 |  |  |
|  | XP\_065067185\_14731-model12 | -49.338 |
| # cluster 13 - Size: 1 |  |  |
|  | XP\_065067185\_14731-model13 | -49.2646 |
| # cluster 14 - Size: 1 |  |  |
|  | XP\_065067185\_14731-model14 | -49.0615 |
| # cluster 15 - Size: 1 |  |  |
|  | XP\_065067185\_14731-model15 | -48.9815 |
| # cluster 16 - Size: 1 |  |  |
|  | XP\_065067185\_14731-model16 | -48.8757 |
| # cluster 17 - Size: 2 |  |  |
|  | XP\_065067185\_14731-model17 | -48.6648 |
|  | XP\_065067185\_14731-model17.1 | -41.7979 |
| # cluster 18 - Size: 1 |  |  |
|  | XP\_065067185\_14731-model18 | -48.1896 |
| # cluster 19 - Size: 1 |  |  |
|  | XP\_065067185\_14731-model19 | -48.0742 |
| # cluster 20 - Size: 1 |  |  |
|  | XP\_065067185\_14731-model20 | -47.8962 |
| # cluster 21 - Size: 1 |  |  |
|  | XP\_065067185\_14731-model21 | -47.446 |
| # cluster 22 - Size: 1 |  |  |
|  | XP\_065067185\_14731-model22 | -47.2858 |
| # cluster 23 - Size: 1 |  |  |
|  | XP\_065067185\_14731-model23 | -47.1171 |
| # cluster 24 - Size: 1 |  |  |
|  | XP\_065067185\_14731-model24 | -46.8578 |
| # cluster 25 - Size: 1 |  |  |
|  | XP\_065067185\_14731-model25 | -46.7844 |
| # cluster 26 - Size: 1 |  |  |
|  | XP\_065067185\_14731-model26 | -46.3671 |
| # cluster 27 - Size: 1 |  |  |
|  | XP\_065067185\_14731-model27 | -46.1579 |
| # cluster 28 - Size: 1 |  |  |
|  | XP\_065067185\_14731-model28 | -46.0826 |
| # cluster 29 - Size: 1 |  |  |
|  | XP\_065067185\_14731-model29 | -46.0039 |
| # cluster 30 - Size: 1 |  |  |
|  | XP\_065067185\_14731-model30 | -45.8797 |
| # cluster 31 - Size: 1 |  |  |
|  | XP\_065067185\_14731-model31 | -45.6591 |
| # cluster 32 - Size: 1 |  |  |
|  | XP\_065067185\_14731-model32 | -45.0566 |
| # cluster 33 - Size: 1 |  |  |
|  | XP\_065067185\_14731-model33 | -44.9009 |
| # cluster 34 - Size: 1 |  |  |
|  | XP\_065067185\_14731-model34 | -44.6832 |
| # cluster 35 - Size: 1 |  |  |
|  | XP\_065067185\_14731-model35 | -44.2269 |
| # cluster 36 - Size: 1 |  |  |
|  | XP\_065067185\_14731-model36 | -44.192 |
| # cluster 37 - Size: 1 |  |  |
|  | XP\_065067185\_14731-model37 | -43.9113 |
| # cluster 38 - Size: 1 |  |  |
|  | XP\_065067185\_14731-model38 | -43.8944 |
| # cluster 39 - Size: 1 |  |  |
|  | XP\_065067185\_14731-model39 | -43.8209 |
| # cluster 40 - Size: 1 |  |  |
|  | XP\_065067185\_14731-model40 | -43.5137 |
| # cluster 41 - Size: 1 |  |  |
|  | XP\_065067185\_14731-model41 | -43.4354 |
| # cluster 42 - Size: 1 |  |  |
|  | XP\_065067185\_14731-model42 | -43.1006 |
| # cluster 43 - Size: 1 |  |  |
|  | XP\_065067185\_14731-model43 | -43.0671 |
| # cluster 44 - Size: 1 |  |  |
|  | XP\_065067185\_14731-model44 | -43.0288 |
| # cluster 45 - Size: 1 |  |  |
|  | XP\_065067185\_14731-model45 | -42.8855 |
| # cluster 46 - Size: 1 |  |  |
|  | XP\_065067185\_14731-model46 | -42.7374 |
| # cluster 47 - Size: 1 |  |  |
|  | XP\_065067185\_14731-model47 | -42.5997 |
| # cluster 48 - Size: 1 |  |  |
|  | XP\_065067185\_14731-model48 | -42.5969 |
| # cluster 49 - Size: 1 |  |  |
|  | XP\_065067185\_14731-model49 | -41.7458 |
| # cluster 50 - Size: 2 |  |  |
|  | XP\_065067185\_14731-model50 | -41.5466 |
|  | XP\_065067185\_14731-model50.1 | -39.9277 |
| # cluster 51 - Size: 1 |  |  |
|  | XP\_065067185\_14731-model51 | -41.2783 |
| # cluster 52 - Size: 1 |  |  |
|  | XP\_065067185\_14731-model52 | -41.0483 |
| # cluster 53 - Size: 1 |  |  |
|  | XP\_065067185\_14731-model53 | -40.9675 |
| # cluster 54 - Size: 1 |  |  |
|  | XP\_065067185\_14731-model54 | -40.9273 |
| # cluster 55 - Size: 1 |  |  |
|  | XP\_065067185\_14731-model55 | -40.8622 |
| # cluster 56 - Size: 1 |  |  |
|  | XP\_065067185\_14731-model56 | -40.6598 |
| # cluster 57 - Size: 1 |  |  |
|  | XP\_065067185\_14731-model57 | -40.6566 |
| # cluster 58 - Size: 1 |  |  |
|  | XP\_065067185\_14731-model58 | -40.5987 |
| # cluster 59 - Size: 1 |  |  |
|  | XP\_065067185\_14731-model59 | -40.4861 |
| # cluster 60 - Size: 2 |  |  |
|  | XP\_065067185\_14731-model60 | -40.2875 |
|  | XP\_065067185\_14731-model60.1 | -38.3425 |
| # cluster 61 - Size: 1 |  |  |
|  | XP\_065067185\_14731-model61 | -40.0018 |
| # cluster 62 - Size: 1 |  |  |
|  | XP\_065067185\_14731-model62 | -39.7147 |
| # cluster 63 - Size: 1 |  |  |
|  | XP\_065067185\_14731-model63 | -39.5331 |
| # cluster 64 - Size: 1 |  |  |
|  | XP\_065067185\_14731-model64 | -39.0673 |
| # cluster 65 - Size: 2 |  |  |
|  | XP\_065067185\_14731-model65 | -38.8672 |
|  | XP\_065067185\_14731-model65.1 | -34.7614 |
| # cluster 66 - Size: 1 |  |  |
|  | XP\_065067185\_14731-model66 | -38.7766 |
| # cluster 67 - Size: 1 |  |  |
|  | XP\_065067185\_14731-model67 | -38.5239 |
| # cluster 68 - Size: 1 |  |  |
|  | XP\_065067185\_14731-model68 | -37.9188 |
| # cluster 69 - Size: 1 |  |  |
|  | XP\_065067185\_14731-model69 | -37.8811 |
| # cluster 70 - Size: 2 |  |  |
|  | XP\_065067185\_14731-model70 | -37.5383 |
|  | XP\_065067185\_14731-model70.1 | -36.4994 |
| # cluster 71 - Size: 1 |  |  |
|  | XP\_065067185\_14731-model71 | -37.1888 |
| # cluster 72 - Size: 1 |  |  |
|  | XP\_065067185\_14731-model72 | -37.1249 |
| # cluster 73 - Size: 1 |  |  |
|  | XP\_065067185\_14731-model73 | -36.9954 |
| # cluster 74 - Size: 1 |  |  |
|  | XP\_065067185\_14731-model74 | -36.7916 |
| # cluster 75 - Size: 2 |  |  |
|  | XP\_065067185\_14731-model75 | -36.5356 |
|  | XP\_065067185\_14731-model75.1 | -36.268 |
| # cluster 76 - Size: 1 |  |  |
|  | XP\_065067185\_14731-model76 | -36.3231 |
| # cluster 77 - Size: 1 |  |  |
|  | XP\_065067185\_14731-model77 | -36.1827 |
| # cluster 78 - Size: 1 |  |  |
|  | XP\_065067185\_14731-model78 | -36.1762 |
| # cluster 79 - Size: 1 |  |  |
|  | XP\_065067185\_14731-model79 | -36.0691 |
| # cluster 80 - Size: 1 |  |  |
|  | XP\_065067185\_14731-model80 | -35.5948 |
| # cluster 81 - Size: 1 |  |  |
|  | XP\_065067185\_14731-model81 | -35.0873 |
| # cluster 82 - Size: 1 |  |  |
|  | XP\_065067185\_14731-model82 | -34.7533 |
| # cluster 83 - Size: 1 |  |  |
|  | XP\_065067185\_14731-model83 | -34.6403 |
| # cluster 84 - Size: 1 |  |  |
|  | XP\_065067185\_14731-model84 | -34.5878 |
| # cluster 85 - Size: 1 |  |  |
|  | XP\_065067185\_14731-model85 | -34.5331 |
| # cluster 86 - Size: 1 |  |  |
|  | XP\_065067185\_14731-model86 | -34.2267 |
| # cluster 87 - Size: 1 |  |  |
|  | XP\_065067185\_14731-model87 | -33.9767 |
| # cluster 88 - Size: 1 |  |  |
|  | XP\_065067185\_14731-model88 | -33.7628 |
| # cluster 89 - Size: 1 |  |  |
|  | XP\_065067185\_14731-model89 | -33.4495 |
| # cluster 90 - Size: 1 |  |  |
|  | XP\_065067185\_14731-model90 | -32.5616 |
| # cluster 91 - Size: 1 |  |  |
|  | XP\_065067185\_14731-model91 | -32.4557 |
| # cluster 92 - Size: 1 |  |  |
|  | XP\_065067185\_14731-model92 | -31.7644 |
| # cluster 93 - Size: 1 |  |  |
|  | XP\_065067185\_14731-model93 | -31.0875 |
|  |  |  |
